# Red Blood Cell Shape and Deformability in Patients With COVID-19 Acute Respiratory Distress Syndrome

**DOI:** 10.3389/fphys.2022.849910

**Published:** 2022-02-28

**Authors:** Michaël Piagnerelli, Julie Vanderelst, Alexandre Rousseau, Daniel Monteyne, David Perez-Morga, Patrick Biston, Karim Zouaoui Boudjeltia

**Affiliations:** ^1^Intensive Care, CHU-Charleroi Marie-Curie, Université Libre de Bruxelles, Charleroi, Belgium; ^2^Experimental Medicine Laboratory, CHU-Charleroi Vésale, ULB 222 Unit, Université Libre de Bruxelles, Montigny-le-Tilleul, Belgium; ^3^Laboratory of Molecular Parasitology, IBMM, Université Libre de Bruxelles, Charleroi, Belgium; ^4^IBMM, Center for Microscopy and Molecular Imaging, Université Libre de Bruxelles, Charleroi, Belgium

**Keywords:** COVID-19, red blood cell, microcirculation, deformability, rheology

## Abstract

**Background:**

Acute respiratory distress syndrome due to coronavirus disease 2019 (COVID-19) is associated with high mortality. Several studies have reported that the microcirculation responds adequately to hypoxia in COVID-19 patients by increasing oxygen availability, in contrast to the inadequate response observed in patients with bacterial sepsis. Red blood cells (RBCs), the key cells for oxygen transport, and notably their rheology, are altered during bacterial sepsis, but few data are available in patients with COVID-19.

**Methods:**

In this prospective, non-interventional study, shape was assessed on admission (or inclusion for the volunteers) using Pearson’s second coefficient of dissymmetry (PCD) on the histogram obtained with a flow cytometer technique. A null value represents a perfect spherical shape. RBC deformability was determined using ektacytometry by the elongation index in relation to the shear stress (0.3 to 50 Pa) applied to the RBC membrane. A higher elongation index indicates greater RBC deformability. Results were compared across groups. Scanning electronic microscopy was performed on RBCs from COVID-19 patients. RBC shape and deformability were also assessed on days 3 and 7 in COVID-19 patients.

**Results:**

Forty-nine ICU patients were included (30 with COVID-19 ARDS and 19 with bacterial sepsis). ARDS was more severe in patients with COVID-19 than in those with sepsis (PaO_2_/FiO_2_ 99 [73–154] vs. 270 [239–295] mmHg *p* < 0.001) and mechanical ventilation was more frequently required (87 vs. 21%; *p* < 0.001). Mortality was significantly higher in COVID-19 patients (15/30 [50%] vs. 4/19 [21%], *p* = 0.046). RBCs were significantly more spherical in septic patients (PCD −0.40 [−0.56; −0.18]) than in healthy volunteers (PCD −0.54 [−0.66; −0.49]) but not than in COVID-19 patients (−0.48 [−0.55; −0.43]). In COVID-19 non-survivors (*n* = 11), sphericity was more marked on day 7 (PCD −0.40 [−0.47; −0.28]) than on day 1 (PCD vs. −0.49 [−0.59; −0.44]); *p* = 0.045. At ICU admission, RBC deformability was altered for all shear stress values studied in septic patients compared to COVID-19 patients and healthy volunteers (maximum elongation index for septic patients: 0.600 [0.594–0.630] vs. 0.646 [0.637–0.653] for COVID-19 patients and 0.640 [0.635–0.650] for healthy volunteers; both *p* < 0.001). In the 18 COVID-19 patients studied for 7 days, RBC deformability did not change over time and was not related to outcome. At day 1, RBCs from COVID-19 patients showed a normal structure on scanning electronic microscopy.

**Conclusion:**

In contrast to the significantly altered shape and decreased deformability in patients with bacterial sepsis, RBCs from severely hypoxemic COVID-19 patients had normal deformability on admission, and this pattern did not change over the first week despite a more spherical shape in non-survivors. As RBCs are the key cell for oxygen transport, this maintenance of normal deformability may contribute to the adequate microcirculatory response to severe hypoxia of the microcirculation that has been observed in these patients.

## Introduction

Since the description of the first cases of patients admitted to the intensive care unit (ICU) for coronavirus disease-19 (COVID-19) in December 2019 in China, the pandemic has affected more than 230 million people worldwide (http:who.int). Despite better knowledge about physiopathology, general management, and potential treatments, mortality remains around 35%, especially in hypoxic patients requiring mechanical ventilation and/or venovenous extracorporeal circulation (COVID-ICU group on behalf of the REVA Network and the COVID-ICU Investigators, 2021; Lorusso et al., 2021; Piagnerelli et al., 2021).

Critically ill patients with COVID-19 are characterized by the presence of extreme hypoxia linked to pulmonary alterations secondary to increased presence of the angiotensin-converting enzyme 2 (ACE2) receptor in the lungs, expressed by alveolar epithelial type 2 cells that serve as a reservoir for viral invasion (Zhang et al., 2020). Moreover, pulmonary vascular endothelialitis with increased angiogenesis leads to an increased rate of immunothrombosis (Ackermann et al., 2020; Piagnerelli et al., 2020) in these patients.

Red blood cells (RBCs), as the oxygen transporters and sensors of local hypoxia, are key elements of the microcirculation (Piagnerelli et al., 2003). Recently, Favaron et al. observed increased oxygen extraction capacity in the microcirculation in COVID-19 patients with severe hypoxia, as a result of increased RBC availability (Favaron et al., 2021). However, although several reports showed alterations in the metabolism and structure of the RBC membrane in COVID-19 patients (Metthew Thomas et al., 2020; Lam et al., 2021; Renoux et al., 2021; Schapkaitz and De Jager, 2021), there are no data available concerning the ability of RBCs to pass through capillaries in COVID-19 ARDS patients and, especially the relationship of the deformability with outcome. For these reasons, we studied changes in RBC shape and deformability in COVID-19 ARDS patients in the ICU compared to ICU patients with sepsis and healthy volunteers.

## Patients and Methods

### Patients

This prospective study was conducted in a 32-bed medico-surgical ICU in the CHU-Charleroi Marie-Curie, Belgium, after approval by the local ethics committee (ISPPC OM 008).

All adult ICU patients with severe COVID-19 during April 2021 were considered for inclusion in this single center study. The inclusion criteria were as follows: ≥18 years; moderate to severe ARDS according to the Berlin definition (The ARDS Definition Task Force, 2012), i.e., a PaO_2_/FiO_2_ ratio < 200 mmHg with a positive end-expiratory pressure (PEEP) of at least 5 mmHg receiving invasive ventilation and bilateral lung infiltrates; and a positive reverse transcriptase-polymerase chain reaction result for severe acute respiratory syndrome-related coronavirus 2 (SARS-CoV-2). We excluded patients with known hemopathy, pregnancy, active bleeding, or active cancer, and transfer from other hospitals.

We also enrolled a group of patients with bacterial sepsis. Sepsis was diagnosed on the basis of the Third International Consensus Definitions for Sepsis and Septic Shock (Singer et al., 2016) with a proven bacterial infection. Finally, we enrolled a group of healthy hospital employees as controls.

The following demographic data were recorded from patients at ICU admission: age, sex, body mass index (BMI), co-morbidities (diabetes mellitus and arterial hypertension), delay between symptoms and ICU admission and between hospital and ICU admission, SAPS III (Moreno et al., 2005) score, need for mechanical ventilation and vasopressors, length of ICU stay, and ICU mortality. We also recorded the following laboratory data: hemoglobin concentration, hematocrit, erythrocyte count, leukocyte count, lactate, and C-reactive protein (CRP) concentrations. The lowest value of PaO_2_/FiO_2_ was also reported. The sequential organ failure assessment (SOFA) score (Vincent et al., 1996) was recorded on day 1 of the ICU admission.

For the COVID-19 group, blood analyses were done at ICU admission, after 3 days and at 7 days. The two other groups had one blood sample taken on inclusion. In septic and COVID-19 patients, blood samples were collected simultaneously with the arterial blood gas analysis.

### Methods

#### Measurements of RBC Shape

As change in RBC shape is time dependent, analyzes were completed in a maximum of 90 min. Measurement techniques have been described elsewhere (Piagnerelli et al., 2003, 2007). Briefly, using this technique the RBC shape of healthy volunteers shows a bimodal distribution related to the ellipsoid form. In many disease states (chronic renal failure, diabetes mellitus, and sepsis), RBCs are characterized by a more spherical shape (Piagnerelli et al., 2007). This technique could facilitate investigation of abnormalities of RBC rheology and be used to monitor RBC shape in various disease states.

Data were collected on a MacsQuant® Analyzer 10 flow cytometer (Miltenyi Biotec BV, ZZ Leiden, Netherlands). The forward light scatter channels (FSCs) were set on a linear scale. Cell size is the principal component of the FSC signal. For estimation of RBC shape, we used the second coefficient of dissymmetry of Pearson (PCD), applying low shear stresses (12 μl/min RBC flow rate) to allow the RBCs to rotate in the flow without deformation (Bitbol, 1986). In this technique, we did not add fluorescently labelled agglutinins that can alter RBC shape.

Whole blood (2 μl) was mixed with isotonic (286 mOsm) phosphate-buffer saline at 25°C. This study was limited to 15,000 events and lasted for 15 s. In healthy volunteers, after positioning of the obscuration bar, the cytometer viewed the flow of ellipsoid, biconcave RBCs as essentially two populations of cells, and the FSC histograms showed a typically bimodal distribution of RBCs.

We calculated the PCD = (3 × [mean − median]/SD) on the histogram as an estimation of the sphericity of the RBCs (Piagnerelli et al., 2003, 2007). In general, the PCD values of RBCs in healthy volunteers are around −0.6 and a PCD value of 0 represents a perfect spherical shape.

#### Measurements of RBC Deformability

RBC deformability was assessed using ektacytometry (LORRCA; Mechatronics Instruments BV, AN Zwaag, Netherlands). We used the same analytical method as that used by Donadello et al. (2015): a suspension of RBCs was mixed with polyvinylpyrrolidone 360 solution, an isotonic viscous medium (PVP, 4%; MW 360 kDa; viscosity 30 ± 2 mPa·s), to obtain a final solution with a constant hematocrit of 0.2%. Using a Couette system composed of a glass cup and a precisely fitting bob, with a gap of 0.36 mm between the cylinders, the liquid solution was sheared and illuminated by a laser beam in order to obtain a diffraction pattern produced by the deformed cells. This diffraction was analyzed by a computer, which also controls the cup rotational speed and the predetermined shear stresses. The elongation index (EI) is calculated as: EI = (L − W)/(L + W), where L and W are the length and width of the diffraction pattern, respectively. The geometry of the diffraction pattern is elliptical. For a given shear stress, the greater the RBC deformability, the higher the EI. At 37°C, we assessed the EI curves for 12 consecutive shear stress values, because human RBC deformability reaches a plateau at 50 Pa: 0.3, 0.48, 0.76, 1.21, 1.93, 3.07, 4.89, 7.78, 12.3, 19.7, 31, and 50 Pa. Interassay variabilities for each shear stress were as: 51, 8.2, 3.9, 2.4, 1.7, 1.6, 1.1, 1.3, 1.7, 1.2, 1.4, and 1.2%, respectively.

From the shear stress-response curves of shape change, we calculated the maximal RBC elongation (EI max) and curves are presented in logarithmic scales (Condon et al., 2003).

#### Scanning Electronic Microscopy Procedure

Whole blood was obtained after venipuncture on tube without anticoagulant. The tube was placed at 37°C during 30 min. Then, clots were washed three times with cacodylate buffer before scanning electronic microscopy procedure. This procedure was performed on three COVID-19 patients.

### Statistical Analysis

The clinical characteristics of the patients are presented as median values [25–75%] or percentages and were compared by the Kruskal-Wallis test for all pairwise multiple comparison procedures (Dunn’s Method) or Mann–Whitney Rank Sum test when two groups were compared. PCD and EI are presented as median values [25–75%]. Comparisons between the three groups were made using a one-way analysis of variance. The change in PCD or EI over time was evaluated using a Friedman repeat measures analysis of variance with Bonferroni post-hoc adjustments. A value of *p* < 0.05 was considered as statistically significant.

## Results

Forty-nine ICU patients were included (30 COVID-19 ARDS patients and 19 septic patients) and 21 volunteers.

Demographic and biological data of the patients are shown in Table 1. Seventeen of the 19 patients with sepsis (89%) were in shock at the moment of inclusion. Bacterial sepsis was due to peritonitis in eight patients (32%), pneumonia in six (32%), meningitides in two (10%), urinary tract infection in two (10%), and osteitis in one (5%); three of these patients (16%) also had a positive blood culture.

**Table 1:** Subject demographics, biological characteristics, and outcomes.

	COVID-19 patients (*n* = 30)	Septic patients (*n* = 19)	Healthy volunteers (*n* = 21)
Age (years)	62 [54–70]	68 [59–74]	53 [45–60]^*^^,^^£^
Men (%)	22 (73)	14 (67)	10 (48)
BMI	33 [29–36]	28 [25–33]	23 [20–25]^*^
Diabetes (%)	10 (33)	5 (26)	NR
Arterial hypertension (%)	11 (37)	11 (58)	NR
Delay symptoms/ ICU admission (days)	7 [5–7]	NR	NR
Delay hospital/ICU admission (days)	1 [0–2]	NR	NR
SAPS III	57 [51–63]	56 [53–69]	NR
SOFA at day 1	4 [2–7]	7 [3–9]	NR
Hemoglobin (g/L)	143 [137–161]^£^	104 [88–135]	132 [121–146]^£^
Hematocrit (%)	40.9 [36.6–42.9]^£^	31.4 [27.1–39.6]	43.6 [40.1–47.0]^£^
RBC count (T/L)	4.5 [4.1–4.8]^£^	3.6 [3.0–4.1]	4.9 [4.3–5.4]^£^
Leukocyte count (G/L)	9.1 [7.1–11.6]	13.3 [9.1–16.7]	6.0 [5.3–6.8]^*^^,^^£^
CRP (mg/L)	91 [50–137]	186 [94–277]	1.0 [0.5–4.0]^*^^,^^£^
Lactate (mmol/L)	1.2 [0.9–1.5]^£^	1.6 [1.3–2.0]	NR
PaO_2_/FiO_2_	99 [73–154]^$^	270 [239–295]	NR
Mechanical ventilation (%)	26 (87)^$^	4 (21)	NR
Vasopressors (%)	2 (7)^$^	17 (89)	NR
Length of ICU stay (days)	12 [6–18]^£^	5 [3–6]	NR
ICU mortality (%)	15 (50)^£^	4 (21)	NR

Results are presented as median [25th-75th] percentiles and compared by Mann–Whitney test. NR, non relevant; BMI, body mass index; CRP, C-reactive protein; RBC, red blood cell; SAPS III, Simplified acute physiology score 3; and SOFA, sequential organ failure assessment.

* *p* < 0.05 vs. COVID-19 patients.

£ *p* < 0.05 vs. septic patients.

$ *p* < 0.001 vs. septic patients.

As expected, PaO_2_/FiO_2_ was lower in COVID-19 patients than in the other group and 26/30 (87%) of these patients needed mechanical ventilation. CRP concentration was significantly higher in septic patients than in the other groups.

The COVID-19 patients had a longer ICU stay than the sepsis patients and greater ICU mortality (50 vs. 21%).

### RBC Shape

The PCD was significantly lower in septic patients than in healthy volunteers (−0.40 [−0.56; −0.18] vs. −0.54 [−0.66; −0.49]; *p* < 0.05); there were no differences in PCD values compared to COVID-19 patients (−0.48 [−0.55; −0.43]). There was no significant difference overall in the PCD in COVID-19 ICU survivors and non-survivors (PCD: −0.48 [−0.55; −0.45] vs. −0.49 [−0.59; −0.44]; *p* = 0.94), but the PCD was significantly lower on day 7 than on day 1 in the non-survivors (*n* = 11; PCD −0.49 [−0.59; −0.44] on day 1 vs. –0.40 [−0.47; −0.28] on day 7; *p* = 0.045).

### RBC Deformability

**Figure 1:**
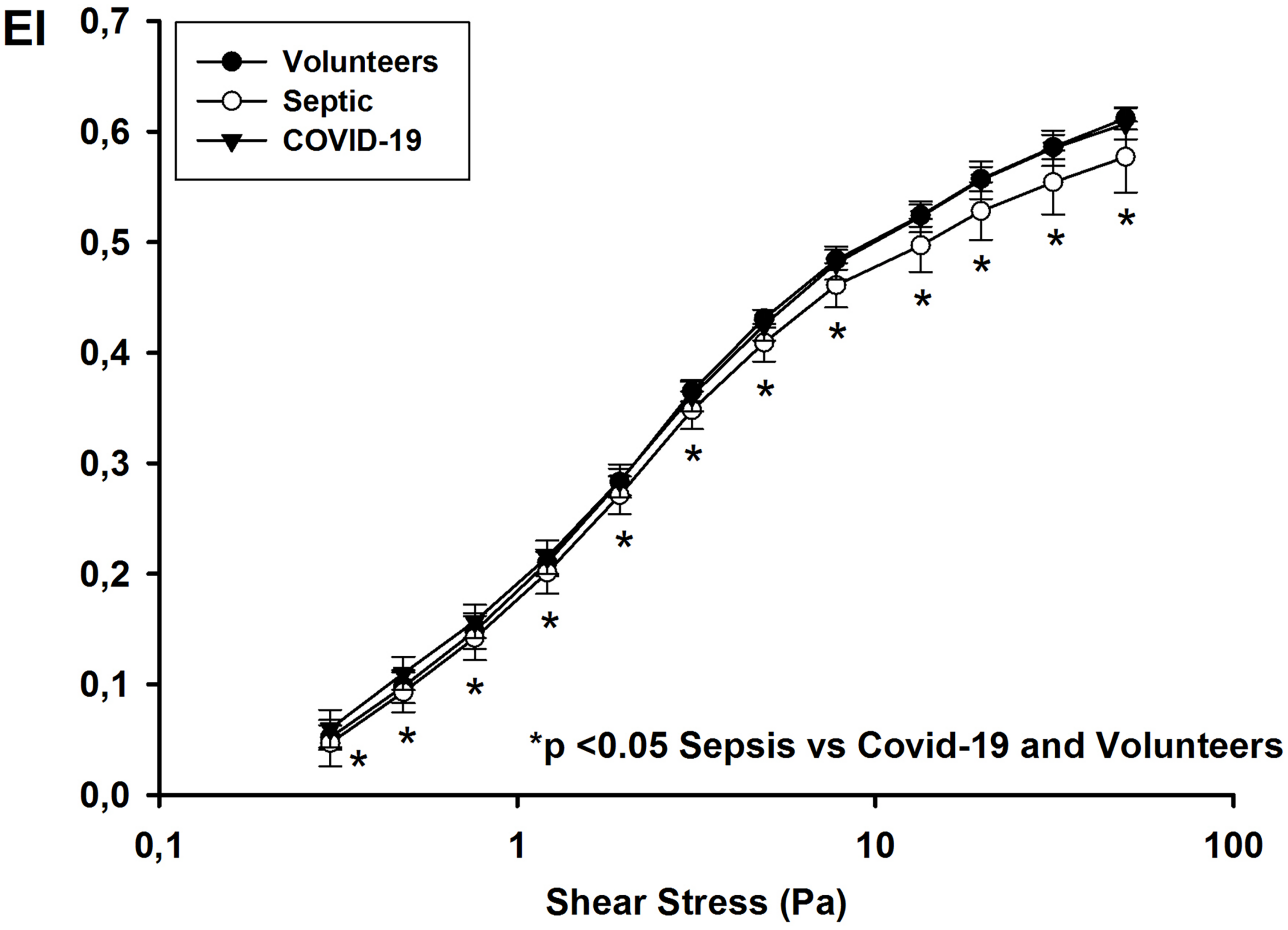
Elongation index at different shear stress values on day 1 in healthy volunteers, and patients with COVID-19 and non-COVID septic.

For all shear stresses studied, the EIs in septic patients were significantly lower than those in COVID-19 patients and healthy volunteers (Figure 1). The EI max for septic patients was 0.600 [0.594–0.630] compared to 0.646 [0.637–0.653] for COVID-19 patients and 0.640 [0.635–0.650] for healthy volunteers (*p* < 0.001). RBC deformability was similar in volunteers and COVID-19 patients and in survivors and non-survivors (EI max for survivors: 0.645 [0.641–0.653] vs. for non-survivors: 0.648 [0.633–0.654]; *p* = 0.62).

**Figure 2:**
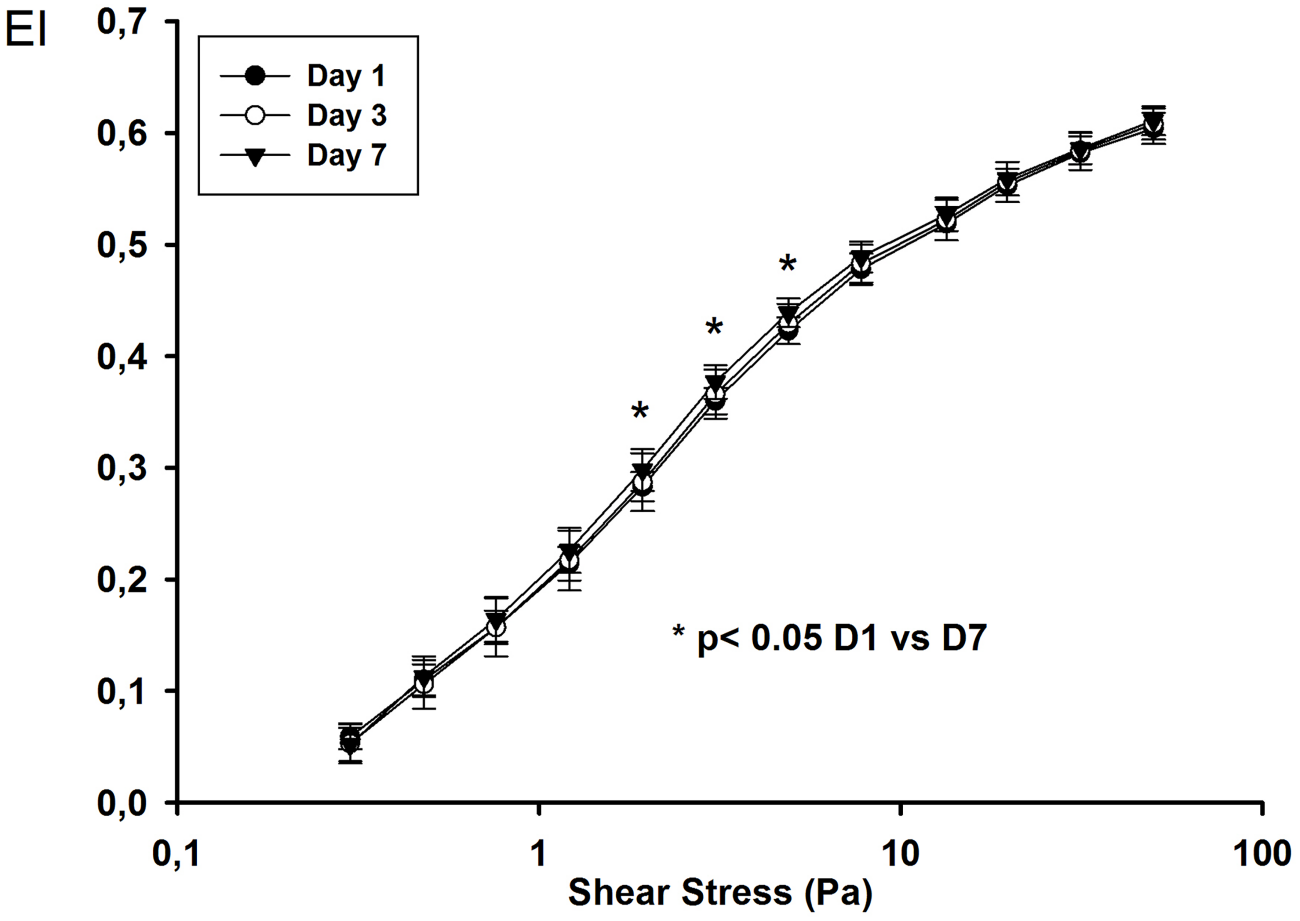
Elongation index at different shear stress values on day 1, day 3, and day 7 in COVID-19 ARDS patients.

We followed 18 ARDS COVID-19 patients until day 7. The EI values were higher on day 7 for intermediate shear stress values (1.93, 3.07, and 4.89 Pa; Figure 2).

### RBC Scanning Electronic Microscopy

**Figure 3:**
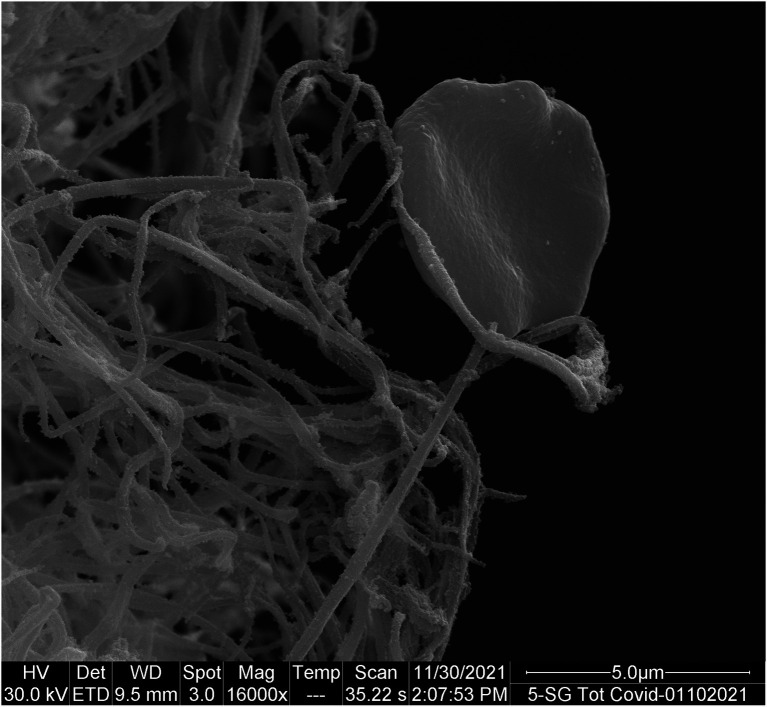
RBC of a COVID-19 patient with a typical biconcave shape.

At day 1, RBCs of COVID-19 patients exhibit a typical biconcave shape without changes in membrane structure (Figure 3).

## Discussion

COVID-19 patients with ARDS, especially non-survivors, showed altered RBC shape as assessed by flow cytometry, but with no change in RBC deformability. Indeed, the RBC deformability was only significantly altered in patients with bacterial sepsis.

Using flow cytometry and ektacytometry, we previously observed altered RBC shape and deformability in septic patients (Piagnerelli et al., 2003; Reggiori et al., 2009; Donadello et al., 2015) and reported that persistence of these alterations was associated with poor outcome (Donadello et al., 2015). We also showed that a more spherical RBC shape in septic patients was associated with decreased sialic acid (SA) content in the RBC membrane (Piagnerelli et al., 2003, 2009) and reproduced these RBC shape alterations in septic patients by incubating RBCs from healthy volunteers with neuraminidase, an enzyme that cleaves SA on the RBC membrane (Piagnerelli et al., 2009). These modifications of SA content and sphericity stimulated anaerobic glycolysis as demonstrated by increased intra-erythrocytic concentrations of 2,3 diphosphoglycerate and lactate (Piagnerelli et al., 2009). These results suggest links between the membrane, where glycolytic enzymes are anchored (Campanella et al., 2005), and an enhanced ability of the altered RBCs to produce and liberate ATP by glycolysis (McMahon et al., 2021). Although RBC SA content was decreased, the membrane proteins were not modified (Piagnerelli et al., 2012), in contrast to the lipid part of the RBC membrane in patients with bacterial sepsis (Kempe et al., 2007). In agreement with these findings, several studies in COVID-19 patients have also reported alterations of the RBC membrane and metabolism. Thomas et al. showed that RBCs from 29 COVID-19 patients had increased levels of glycolytic intermediates, accompanied by oxidation and fragmentation of several proteins (ankyrin, spectrin beta, and the cytosolic domain of band 3; Thomas et al., 2020). They also observed altered lipid metabolism (short- and medium-chain saturated fatty acids, acyl-carnitines, and sphingolipids). No data on the severity of the COVID-19 disease in these patients were provided and no attempts were made to analyze relationships between metabolism and RBC shape or deformability.

RBCs from COVID-19 patients become more spherical over time but this change was only significant on day 7 in non-survivors, suggesting severe alterations of shape in these patients. As the flow cytometry technique assesses RBC shape, these alterations may be due to deposits on the surface of the RBC membrane. Indeed, Lam et al. observed an increased deposit of complement activation proteins (C3/C3b/iC3b/C3dg + and C4d) on the membrane of RBCs from 72 COVID-19 patients compared to healthy control subjects, but the amount of deposit was similar in 11 patients with non-COVID sepsis (Lam et al., 2021). Interestingly, the complement protein deposit on RBCs from the COVID-19 patients increased significantly over time. No correlations with mortality (18% in the COVID-19 patients enrolled) were made in this study (Lam et al., 2021). We can speculate that our results of a significantly more spherical shape at day 7 only in patients with poor outcome may therefore be related to a larger deposit of complement proteins on the RBC surface membrane in more severe COVID-19 disease.

The cytometry technique used in this study only enables estimation of the RBC shape and not the deformability capacity, the major determinant allowing RBCs to pass through the capillaries to exchange oxygen to the cells. Recently, Renoux et al. (2021) reported a significantly lower RBC deformability in seven COVID-19 patients for shear stress values from 1.69 to 30 Pa compared to healthy volunteers. These authors also observed a lower deformability in seven septic patients compared to healthy volunteers but only for a shear stress of 30 Pa (Renoux et al., 2021). The difference with our results may be explained by the number of patients studied and differences in the characteristics of the included patients. In contrast to Renoux et al., we only included severely ill COVID-19 and septic patients, all of whom were admitted to the ICU, with a high rate of mechanical ventilation, and with shock for those with sepsis. No data on severity were provided in the study by Renoux et al., but the patients were probably less critically ill (with no deaths in the COVID-19 and sepsis groups, and just three of the seven COVID-19 patients admitted to the ICU).

Our results can be added to the results of Favaron et al. who studied the response of the microcirculation to hypoxemia in COVID-19 ARDS patients on the ICU. These authors observed increased oxygen extraction capacity by increased RBC availability (increased total vessel density, functional capillary density, proportion of perfused vessels, RBC velocity, capillary hematocrit, and capillary-hematocrit-to-systemic-hematocrit ratio; Favaron et al., 2021). These modifications of the microcirculation were similar to those that occur in healthy volunteers during exposure to hypoxia at high altitude (D’Alessandro et al., 2016). Indeed, D’Alessandro et al. (2016) also observed, in RBCs from healthy volunteers after exposure to high altitude (>5,000 m), a competitive binding of deoxyhemoglobin and glycolytic enzymes to the N-terminal cytosolic domain of band 3. These modifications promote the accumulation of 2,3-diphosphoglycerate, stabilizing the deoxygenated state of hemoglobin, and cytosolic acidification, triggering oxygen off-loading through the Bohr effect. As a response mechanism to severe hypoxia, RBCs from COVID-19 patients also increase concentrations of 2,3-diphosphoglycerate, favoring hemoglobin-oxygen off-loading as demonstrated by Thomas et al.

There are several limitations to our study. First, we only measured RBC shape and deformability, and relationships with metabolism and alterations of the membrane remain speculative. Second, we only studied ICU patients and the time course of these alterations before ICU admission may be important. This effect, however, may be limited because COVID-19 ARDS patients were rapidly admitted to the ICU (delay hospital/ICU admission of 1 [0–2] days). Third, only two of the patients in the sepsis group had ARDS and could limit the role of hypoxia on RBC deformability. Four, we did not estimate volemia in ICU septic and COVID-19 patients, which may influence RBC rheology. The effect must be limited because studies on RBCs were performed as soon as these patients are ICU admitted. Moreover, previous studies do not show effects of either blood osmolality on RBC shape (Piagnerelli et al., 2003), or a correlation between anemia and RBC shape and deformability (Piagnerelli et al., 2003; Reggiori et al., 2009).

In conclusion, our results suggest that the RBCs of COVID-19 patients with severe hypoxemia have a normal deformability on admission, and this pattern did not change over the first week despite a more spherical shape in non-survivors. This pattern is an additional element in an adequate response of the microcirculation to the hypoxia observed in these patients.

## Data Availability Statement

The raw data supporting the conclusions of this article will be made available by the authors, without undue reservation.

## Ethics Statement

The studies involving human participants were reviewed and approved by ISPPC OM 008. Written informed consent for participation was not required for this study in accordance with the national legislation and the institutional requirements.

## Author Contributions

MP, JV, and KZ designed the study and wrote the paper. MP, JV, AR, DM, DP-M, PB, and KZ performed the research. All the authors revised the paper critically and approved the final version.

## Conflict of Interest

The authors declare that the research was conducted in the absence of any commercial or financial relationships that could be construed as a potential conflict of interest.

## Publisher’s Note

All claims expressed in this article are solely those of the authors and do not necessarily represent those of their affiliated organizations, or those of the publisher, the editors and the reviewers. Any product that may be evaluated in this article, or claim that may be made by its manufacturer, is not guaranteed or endorsed by the publisher.
